# Occasional cannabis use is associated with higher premorbid functioning and IQ in youth at clinical high-risk (CHR) for psychosis: Parallel findings to psychosis cohorts

**DOI:** 10.1016/j.schres.2024.07.032

**Published:** 2024-07-30

**Authors:** L. Kennedy, B.S. Ku, J. Addington, C.M. Amir, C.E. Bearden, T.D. Cannon, R. Carrión, B. Cornblatt, M. Keshavan, D. Perkins, D. Mathalon, W. Stone, E. Walker, S. Woods, K. S. Cadenhead

**Affiliations:** a Department of Psychiatry, University of California San Diego, United States; b Department of Psychiatry and Behavioral Sciences, Emory University School of Medicine, Atlanta, GA, United States; c University of Calgary, Calgary, Canada; d Semel Institute for Neuroscience and Human Behavior, Department of Psychiatry, University of California Los Angeles, Los Angeles, CA, United States; e Department of Psychology, Yale University, New Haven, CT, United States; f Department of Psychiatry, Donald and Barbara Zucker School of Medicine at Hofstra/Northwell, Hempstead, NY, United States; g Beth Israel Deaconess Medical Center and Harvard Medical School, Boston, MA, United States; h University of North Carolina, Chapel Hill, Chapel Hill, NC, United States; i Department of Psychiatry and Behavioral Sciences, Weill Institute for Neurosciences, University of California, San Francisco; j Department of Psychiatry, Yale University, New Haven, CT, United States

**Keywords:** Clinical high-risk (CHR) psychosis, Neurocognition, Cannabis, Functioning

## Abstract

**Background:**

Neurocognitive deficits have been widely reported in clinical high-risk for psychosis (CHR) populations. Additionally, rates of cannabis use are high among CHR youth and are associated with greater symptom severity. Cannabis use has been sometimes shown to be associated with better neurocognition in more progressed psychosis cohorts, therefore in this study we aimed to determine whether a similar pattern was present in CHR.

**Methods:**

CHR participants ages 12–30 from the North American Prodromal Longitudinal Study (NAPLS-3) (*N* = 698) were grouped according to: “minimal to no cannabis use” (*n* = 406), “occasional use” (*n* = 127), or “frequent use” (*n* = 165). At baseline, cannabis use groups were compared on neurocognitive tests, clinical, and functional measures. Follow-up analyses were used to model relationships between cannabis use frequency, neurocognition, premorbid, and social functioning.

**Results:**

Occasional cannabis users performed significantly better than other use-groups on measures of IQ, with similar trend-level patterns observed across neurocognitive domains. Occasional cannabis users demonstrated better social, global, and premorbid functioning compared to the other use-groups and less severe symptoms compared to the frequent use group. Follow-up structural equation modeling/path analyses found significant positive associations between premorbid functioning, social functioning, and IQ, which in turn was associated with occasional cannabis use frequency.

**Discussion:**

Better premorbid functioning positively predicts both better social functioning and higher IQ which in turn is associated with a moderate cannabis use pattern in CHR, similar to reports in first-episode and chronic psychosis samples. Better premorbid functioning likely represents a protective factor in the CHR population and predicts a better functional outcome.

## 1. Introduction

Rates of cannabis use are higher among young people at clinical high-risk for developing psychosis (CHR) compared to healthy counterparts, and are associated with risk for conversion to a psychotic disorder in some (Di Forti et al., 2014; Kraan et al., 2016; Kristensen and Cadenhead, 2007; Marconi et al., 2016; Valmaggia et al., 2014; van der Meer et al., 2012; van der Steur et al., 2020) but not all (Addington et al., 2014; Auther et al., 2012, 2015; Buchy et al., 2014; Carney et al., 2017; Phillips et al., 2002; Santesteban-Echarri et al., 2022) studies. Cannabis use also appears to exacerbate positive and negative attenuated psychosis symptoms (Amir et al., 2022; Colizzi and Murray, 2018; Marconi et al., 2016; Santesteban-Echarri et al., 2022; van der Meer et al., 2012) and may impact functional outcomes in CHR however findings have been inconsistent (Carrión et al., 2023; Chester et al., 2023; West and Sharif, 2023).

While studying cannabis use in CHR is of critical importance in the context of symptoms and conversion to psychosis, it is also important to evaluate how cannabis use may be associated with other domains that affect long-term functioning; specifically, neurocognition. Significant impairment in cognition and a decline in functioning are considered primary features of psychotic disorders (Bowie and Harvey, 2006; Fulford et al., 2013; Rabin et al., 2011). Neurocognitive impairments are well-documented across several domains in psychosis populations, including working memory, sustained attention, processing speed, and executive functioning (Kalkstein et al., 2010; Lepage et al., 2014; Stone and Seidman, 2016). As early as the premorbid and CHR phases of illness, individuals display notable impairments across similar neurocognitive domains (Seidman et al., 2016b). Previous retrospective studies of individuals who later developed psychosis-spectrum disorders note that cognitive dysfunction, such as lower intelligence quotient (IQ), was present in early childhood prior to the onset of CHR symptoms (Seidman et al., 2013). Studies among individuals who later developed a psychotic disorder also suggest that premorbid neurocognitive impairment was on par to that observed in first-degree relatives of patients with psychosis (Giuliano et al., 2012; Karcher et al., 2023; Keshavan et al., 2010; Stone and Seidman, 2016). In CHR cohorts, neurocognitive deficits in visual and verbal learning, working memory, processing speed, and attention comprise the most significant liability for a later transition to psychosis (Catalan et al., 2021). Deterioration in social and global functioning are also observed beginning in the premorbid period and are associated with symptoms and impairments in neurocognition in addition to being harbingers of future clinical outcomes in early psychosis populations (Carrión et al., 2021; Cornblatt et al., 2012; Fulford et al., 2013; Lyngberg et al., 2015; Mensi et al., 2023; Seidman et al., 2016b).

The association of cannabis use with neurocognition and functioning has primarily been examined in patients with psychosis and in the general population with mixed results in both samples (Albaugh et al., 2023; Bogaty et al., 2018; Colizzi et al., 2020; Colizzi and Murray, 2018; Dellazizzo et al., 2022, 2023; Sanchez-Gutiérrez et al., 2020; Volkow et al., 2016). A recent systematic review by Dellazizzo et al. (2023) highlighted several studies among psychosis cohorts in which cannabis users perform better across neurocognitive measures compared to non-users, however this was not consistent across studies and findings appeared to be domain specific. Meta-analyses of healthy non-psychiatric adolescents have shown strong associations between repeated cannabis use, altered brain structure and impairments in cognitive functioning (Dellazizzo et al., 2022; Jacobus and Tapert, 2014; Lisdahl et al., 2014; Meier et al., 2012; Scott et al., 2018). Importantly, there remains a debate as to any causal relationship between cannabis use and neurocognitive deficits in healthy youth because some studies have suggested that neurocognition may predict later cannabis use (Dellazizzo et al., 2022; Scott et al., 2018). Less is known however about the potential effect of cannabis on neurocognition and associated functioning in the younger, more heterogeneous CHR population (Farris et al., 2020a; West and Sharif, 2023) who are neurocognitively and functionally vulnerable but not psychotic (Catalan et al., 2021; Pedruzo et al., 2023).

Similar to psychosis cohorts, recent reviews of the handful of studies in CHR cohorts have highlighted that findings regarding cannabis use and neurocognition are varied (Farris et al., 2020b; Hasan et al., 2020; West and Sharif, 2023). Among cross-sectional studies of CHR youth, some researchers have demonstrated that cannabis use is associated with domain specific impairments in cognition such as processing speed, executive functioning, and attention (Mensi et al., 2023). Other studies of CHR cohorts found no apparent differences in neurocognitive performance between cannabis users and non-users (Buchy et al., 2015; Bugra et al., 2013; Korver et al., 2010). In contrast, a recent study from the NAPLS-3 cohort comparing CHR youth to youth with 22.q Deletion Syndrome (a genetically high risk for psychosis population) demonstrated a positive association between IQ and cannabis use frequency among the CHR group (Amir et al., 2022). Carrión et al. (2023) found that across two years, CHR youth who endorsed “continuous” and “occasional” recreational cannabis use demonstrated improvements in global neurocognition and functioning over time, suggesting the presence of a unique functional profile of CHR who use cannabis given that CHR youth in general typically display notable impairments in this domain (Addington et al., 2008; Ballon et al., 2007).

In response to the mixed findings regarding the cannabis-neurocognition-functioning paradigm in CHR and in patients with psychosis, we aimed to investigate the relationship of cannabis use to neurocognitive, functional, and clinical domains in a large population of CHR participants from the third iteration of the North American Prodromal Longitudinal Study (NAPLS-3). Based on previous findings of a possible dose-response association between cannabis use, positive symptoms, and psychosis-risk (Di Forti et al., 2014; Farris et al., 2020b; Marconi et al., 2016; Rabin et al., 2011; Santesteban-Echarri et al., 2022), we also aimed to explore how frequency of cannabis use impacts neurocognition and functioning in CHR.

## 2. Methods

### 2.1. Participants

Participants were recruited from 9 sites across North America as part of the NAPLS3 study. The total sample was comprised of CHR youth (*N* = 699) between the ages of 12–30 as detailed by Addington and colleagues (Addington et al., 2022). Eligible CHR participants met criteria for Attenuated Positive Symptom Syndrome (APSS) based on the Structured Interview for Psychosis-Risk Syndrome (SIPS) (McGlashan et al., 2010). Participants were excluded upon screening if they met criteria for a current or past diagnosis of a psychotic disorder through the Structured Clinical Interview for DSM-5 Disorders (SCID-5), had an IQ < 70, had ever been diagnosed with a central nervous system disorder; or if the APSS were better accounted for by the presence of a psychotic disorder or a cannabis use disorder (First, 2015).

### 2.2. Measures

#### 2.2.1. Substance use

Participants were assessed at baseline and follow-up visits for a total of 2 years (Addington et al., 2022). For this project, only baseline measures and data were included in analyses. Initial APSS criteria was assessed via the SIPS across domains of positive, negative, disorganized, and general symptoms, and symptom severity was measured with the Scale of Psychosis-Risk Symptoms (SOPS) (McGlashan et al., 2010). Cannabis use frequency was determined through both the Alcohol and Drug Use Questionnaire and the Cannabis use Questionnaire (CUQ), two self-report instruments used throughout the NAPLS 1–3 studies (Addington et al., 2007, 2012, 2022; Arseneault et al., 2002; Drake et al., 1996). Participants were subsequently grouped according to frequency of cannabis use (current and greatest past frequency): never or a few times – “minimal to no use”, monthly to yearly – “occasional use”, or daily to weekly use – “frequent use”. These groupings were developed based on total distributions from the overall sample. The daily use groups were combined with those who reported using weekly on the CUQ to form the frequent use group. The CUQ also asked participants the age of their first use of cannabis. Cumulative lifetime use was not recorded in this study, however the CUQ did assess cumulative use in the past 6 months. In this project, highest lifetime cannabis use frequency was defined as the highest reported cannabis use pattern in present or past, and current cannabis use was definedas the reported use-frequency at baseline. Because the time-period for past cannabis use was not clearly defined in this young population, we chose to focus primarily on lifetime use-pattern. Acute intoxication with cannabis was not directly measured as part of this study given that THC can remain detectable for up to a month after last use, however site administrators would use clinical judgment and delay the date of research participation if participants appeared intoxicated or reported use on the day of assessment. Information on frequency and severity of alcohol and tobacco use was also collected as part of the Alcohol and Drug Use Questionnaire (Drake et al., 1996).

#### 2.2.2. Functioning

Lifetime domains of premorbid functioning (early childhood (up to age 11), early adolescence (ages 12–15), late adolescence (ages 16–18), adult (age 19 and above) and total) were assessed through the Premorbid Adjustment Scale (PAS) (Cannon-Spoor et al., 1982; Shapiro et al., 2009). Scores for each domain range on a Likert scale from 0 to 6 where 0 indicates healthy functioning and 6 indicates psychopathology/subsequent dysfunction; higher scores indicate worse premorbid functioning. Each domain includes information regarding social, sociosexual, and academic functioning which are incorporated into the overall domain score. Domain scores are calculated by dividing the score received by the total score possible, and total premorbid functioning is calculated by averaging the scores received in each domain, with all final scores ranging from 0.0 to 1.0 producing a cumulative score to reflect all collective premorbid domains for each participant (Cannon-Spoor et al., 1982; van Mastrigt and Addington, 2002). Importantly, given the age range of included participants in this study, some individuals may have not received scores for some premorbid domains however all subjects received a total score which was an average of the available domain scores. Social and role functioning were evaluated with the Global Functioning-Social and Role Scales, two independent scales that range from 1 to 10 with 10 indicating excellent role or social functioning and 1 indicating extreme social isolation and/or role dysfunction (Carrión et al., 2019; Cornblatt et al., 2007). Global functioning was determined via the Global Assessment of Functioning (GAF) with scores ranging from 0 to 100, with 100 indicating highest possible global functioning (Jones et al., 1995).

#### 2.2.3. Neurocognitive battery

Participants also completed a neuropsychological battery at baseline (Addington et al., 2022). Full-scale IQ estimates were obtained through the Weschler Abbreviated Scale of Intelligence-Second Edition (WASI-II), and estimates of premorbid IQ were obtained via the Wide Range Achievement Test-4 Reading Subtest (WRAT-4: Reading Subtest) (Seidman et al., 2016a; Wechsler Abbreviated Scale of Intelligence–Second Edition). Letter-Number Span (LNS), Hopkins Verbal Learning Test-Revised (HVLT-R), and Brief Assessment of Cognition (BACS): Symbol Coding (BACS SC), subscales of the MATRICS Consensus Cognitive Battery (MCCB) were administered at baseline to assess working memory, verbal learning, and processing speed respectively (Nuechterlein et al., 2008). The Seidman Auditory Continuous Performance Task (CPT) and the Social CPT- Identical Pairs version (CPT-IP) were administered to evaluate auditory-verbal and visual-social vigilance and attention, respectively (Cornblatt et al., 1988; Seidman et al., 2012). The Social CPT-IP assesses sustained attention for shapes, digits, and faces, and provides accuracy scores (i.e., d-prime) in each of these subdomains (Addington et al., 2022).

### 2.3. Statistical analyses

Statistical analyses were performed using SPSS version 28 and MPlus version 8.8. To evaluate group differences in baseline demographic variables, symptoms, global, role, social and premorbid functioning as well as neurocognition across CHR cannabis use frequency groups, univariate analyses of variance (ANOVA) were conducted with post-hoc *t*-tests to compare marginal means. For the analyses of neurocognition, both current and highest lifetime cannabis use frequency patterns were assessed. Primary analyses of neurocognition used test raw scores as the dependent variables with age as a covariate and sex as a within group factor. Given the known impact of socioeconomic status (SES), comorbid alcohol and tobacco use, age of onset of cannabis use, and cumulative cannabis use on cognition as well as observed differences in these variables between cannabis use frequency groups, follow up analyses of neurocognition included these variables as additional covariates/within group factors (Amoretti et al., 2021; Buckley et al., 2019; Czepielewski et al., 2022; Lees et al., 2020; Quigley and MacCabe, 2019; Scott et al., 2018). Preliminary covariate tests conducted including and removing years of education made no statistical impact on the results, so years of education were omitted as a covariate. Bonferroni correction was used to control for multiple comparisons. The family-wise error rates were calculated for analyses of both current and lifetime neurocognition (1-(1–0.05)^13^ = 0.51). Bonferroni correction was used to control the family-wise error set at alpha = 0.05/13 (critical alpha/number of comparisons) for a *p*-value significance threshold of *p* = .004.

MPlus was used to conduct follow-up structural equation modeling and path analyses (SEM/PA) using maximum likelihood estimation (ML) to explore the direct and indirect effects of premorbid functioning (primary exogenous variable), social functioning (exogenous variable), and neurocognition (WRAT-4 Reading Score) (exogenous variable) on lifetime cannabis use frequency (endogenous variable) (Beran and Violato, 2010). WRAT-4 Reading Score and social functioning were used as mediators in the models given observed statistically significant group effects of cannabis use group frequency on these measures in univariate analyses described in the Results section. Rationale for the selection of appropriate regression analyses are described in Supplementary Section 1. Binary logistic regressions were performed employing bootstrapping with 5000 iterations to derive indirect effects and associated *p*-values. For the binary logistic regression, the cannabis use frequency variable was transformed to allow for 2 binary variables indicating a comparison between 1) occasional users and frequent users and 2) occasional users and minimal to no users. Chi Square tests (non-significant tests at alpha = 0.05), CFI and TLI values >0.90, and RMSEA and SRMR values <0.08 were all indices used to indicate adequate model fit. These statistics were interpreted together such that a single index out of range does not negate overall model fit if other indices suggest good fit (Hu and Bentler, 1999; McNeish and Wolf, 2023). For the comparisons between the neurocognitive variables as well as the social functioning variables and cannabis use frequency, unstandardized logistic regression coefficients were interpreted via odds ratios/ML estimation (Sperandei, 2014).

## 3. Results

### 3.1. CHR cannabis use patterns

In the formation of the cannabis use groups, the majority of participants reported no/minimal use or occasional use (monthly-yearly) and relatively few reported daily to weekly use (Table 1). Table 1 outlines CHR baseline demographics by lifetime cannabis use group. There were significant group differences in sex distribution with more females in the occasional use group. The rate of alcohol, tobacco and cumulative cannabis use was higher, age of first use was younger, and family income was lower in the frequent use group. The minimal use group was younger and had fewer years of education.

### 3.2. CHR baseline clinical symptoms and functioning by cannabis use frequency

**Fig. 1.**
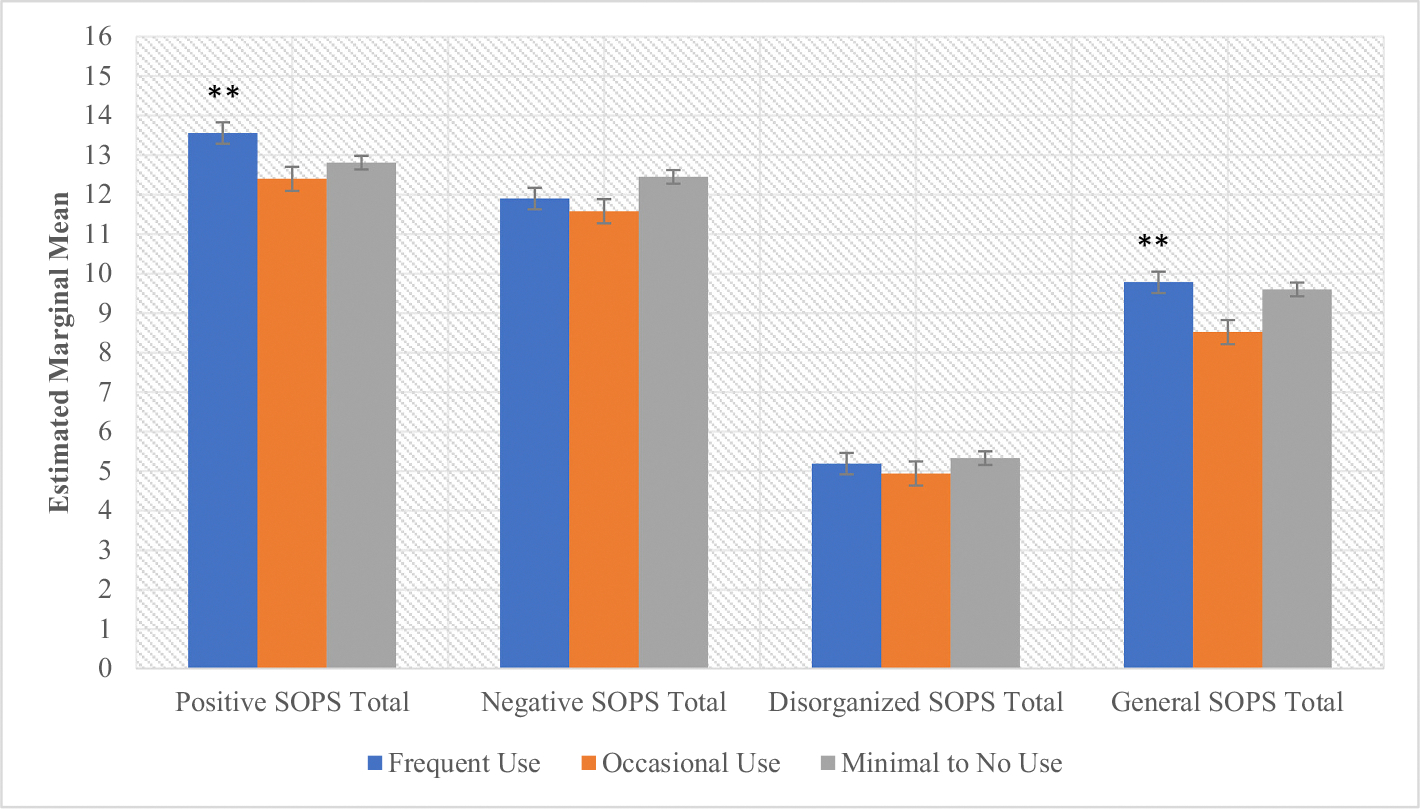
CHR SOPS domains by cannabis use frequency. **p < .05. There was a significant main effect of lifetime cannabis use frequency on SOPS Positive (*F* (2,694) = 4.62, *p* = .01) and General (*F* (2,684) = 4.11, *p* = .02) symptoms. Frequent Users displayed more severe positive symptoms than Occasional users (Mean difference = 1.16, s.e. = 0.40, *p* = .004) and Minimal to No users (Mean difference = 0.77, s.e. = 0.33 *p* = .02). Frequent users also displayed more severe general symptoms than Occasional users (Mean difference = 1.24, s.e = 0.51, *p* = .02). SOPS = Scale of Prodromal Symptoms.

There was a significant main effect of cannabis use frequency on baseline attenuated positive symptoms (*F* = 4.62, *p* = .01) (Fig. 1, Supplementary Table 1). Post-hoc tests revealed that frequent users displayed more severe positive symptoms compared to both lower-use groups (*ps* < .05). Follow-up analyses of individual types of positive symptoms revealed significant group differences by use frequency in grandiosity and perceptual abnormalities; post-hoc tests demonstrated that frequent users displayed more severe grandiose symptoms (*p* < .03) compared to the other use groups) and perceptual abnormalities (*p* < .003) compared to the occasional use group (Supplementary Table 1). By contrast, occasional users reported the least severe perceptual abnormalities compared to both the frequent (*p* < .003) and the minimal to no use groups (*p* = .07) (trend-level). There was a significant main effect of cannabis use frequency and sex (*F* = 4.11, *p* = .02) on current general symptoms with post-hoc tests also demonstrating that general symptoms were more severe among frequent users (*ps* < .05) compared to the other use groups, and females displayed more severe general symptoms compared to males (*p* < .05) (Fig. 1, Supplementary Table 1). No statistically significant interaction effects were observed in analyses of attenuated psychosis symptoms.

**Fig. 2.**
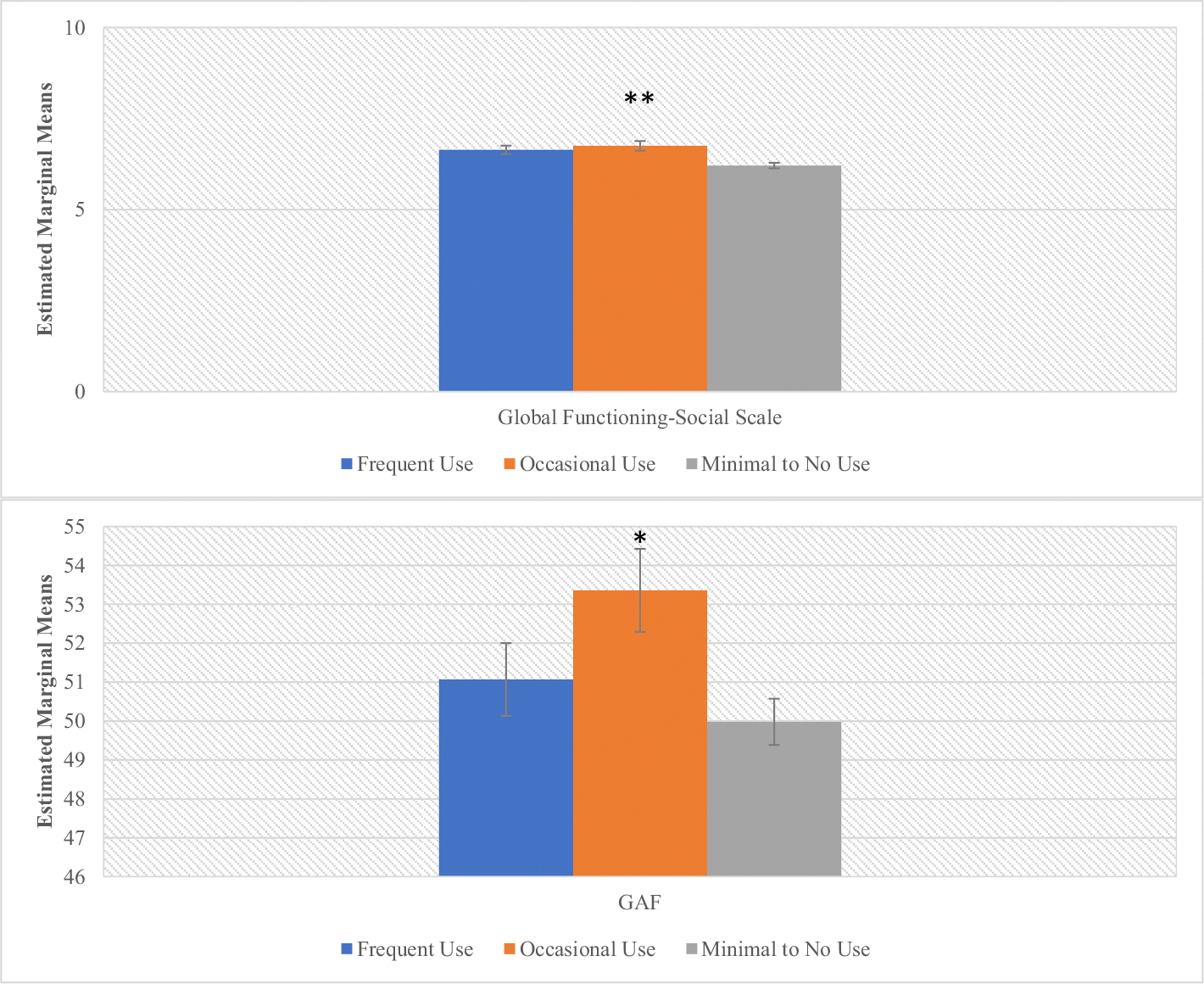
CHR Social and global functioning by cannabis use frequency. **p < .001, *p < .05. There was a significant main effect of cannabis use frequency on social functioning (F (2,694) = 12.29, *p* < .001) with Occasional users displaying significantly higher social functioning than the Frequent (Mean difference = 3.09, s.e. = 1.18, *p* = .06) (trend level) and Minimal to No use (Mean difference = 0.65, s.e = 0.16, *p* < .001) groups. There was also a significant main effect of cannabis use frequency on global functioning (F (2,693) = 3.87, *p* = .02) with Occasional users displaying significantly higher global functioning than the Minimal to No use (Mean difference = −0.38, s.e. = 1.22, *p* = .01) group. GAF = Global Assessment of Functioning.

**Fig. 3.**
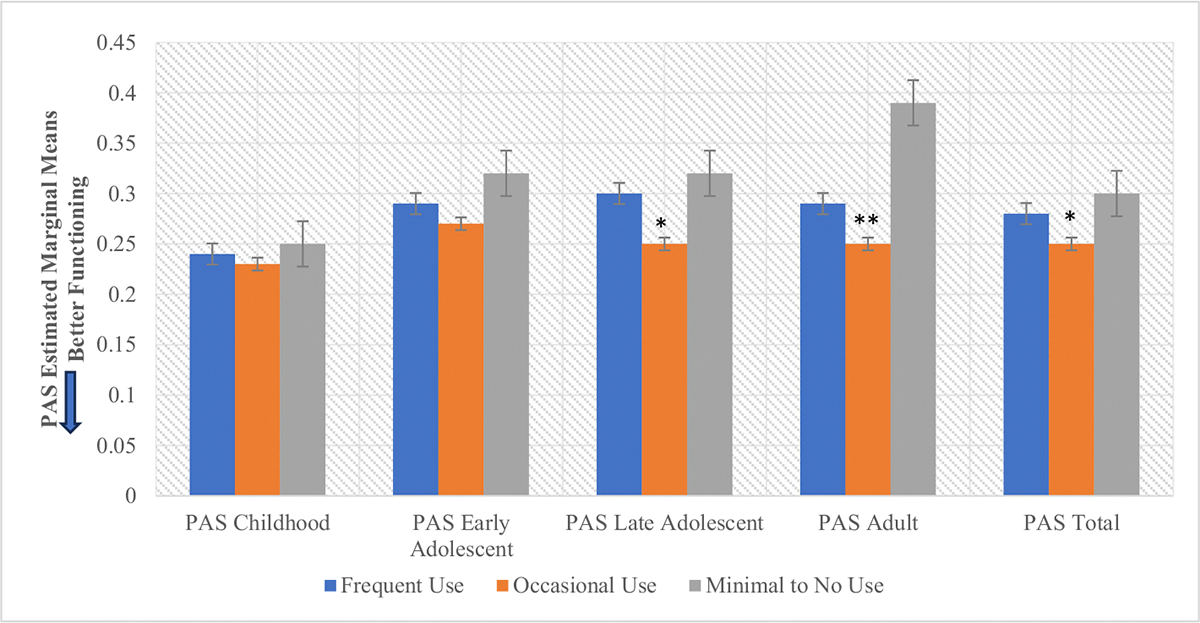
CHR Premorbid functioning domains by cannabis use frequency. *p < .05, **p < .001. There were significant main effects of lifetime cannabis use frequency on Late Adolescent (*F* (2,487) = 5.61, *p* = .004), Adult (*F* (2,253) = 11.35, *p* < .001) and Total (*F*(2, 685) = 4.15, *p* = .02) with Occasional users displaying better premorbid functioning across those three domains. For Late Adolescent premorbid functioning, this relationship was significant (*p* < .05) for the comparison between Occasional users and the other two use groups. For Adult premorbid functioning this relationship was significant (*p* < .001) between the Occasional users and the Minimal to No use groups. For total premorbid functioning, this relationship was significant (*p* < .05) between the Occasional users and the Minimal to No use group. PAS = Premorbid Adjustment Scale.

There was also a significant main effect of cannabis use frequency on baseline social and global functioning (*F* = 12.29, *p* ≤ 0.001, *F* = 3.87, *p* = .02 respectively) (Fig. 2, Supplementary Table 2). Post-hoc analyses showed that occasional cannabis users demonstrated better social (*p* < .001) and global (*p* = .01) functioning at baseline compared to the minimal/no use group (Supplementary Table 2). There was no significant effect of cannabis use frequency on role functioning (*F* = 0.33, *p* = .72). There were significant main effects of cannabis use frequency on total premorbid functioning (*F* = 4.15, *p* = .02) that appeared to be driven by differences in the late adolescent (*F* = 5.61, *p* = .004) and adult premorbid functioning (*F* = 11.35, *p* ≤0.001) categories (meaning that this relationship was significant among older participants), with occasional users (*ps* < .004) displaying better PAS scores across those domains compared to other use groups (Fig. 3, Supplementary Table 3).

### 3.3. CHR Neurocognition by cannabis use frequency

**Fig. 4.**
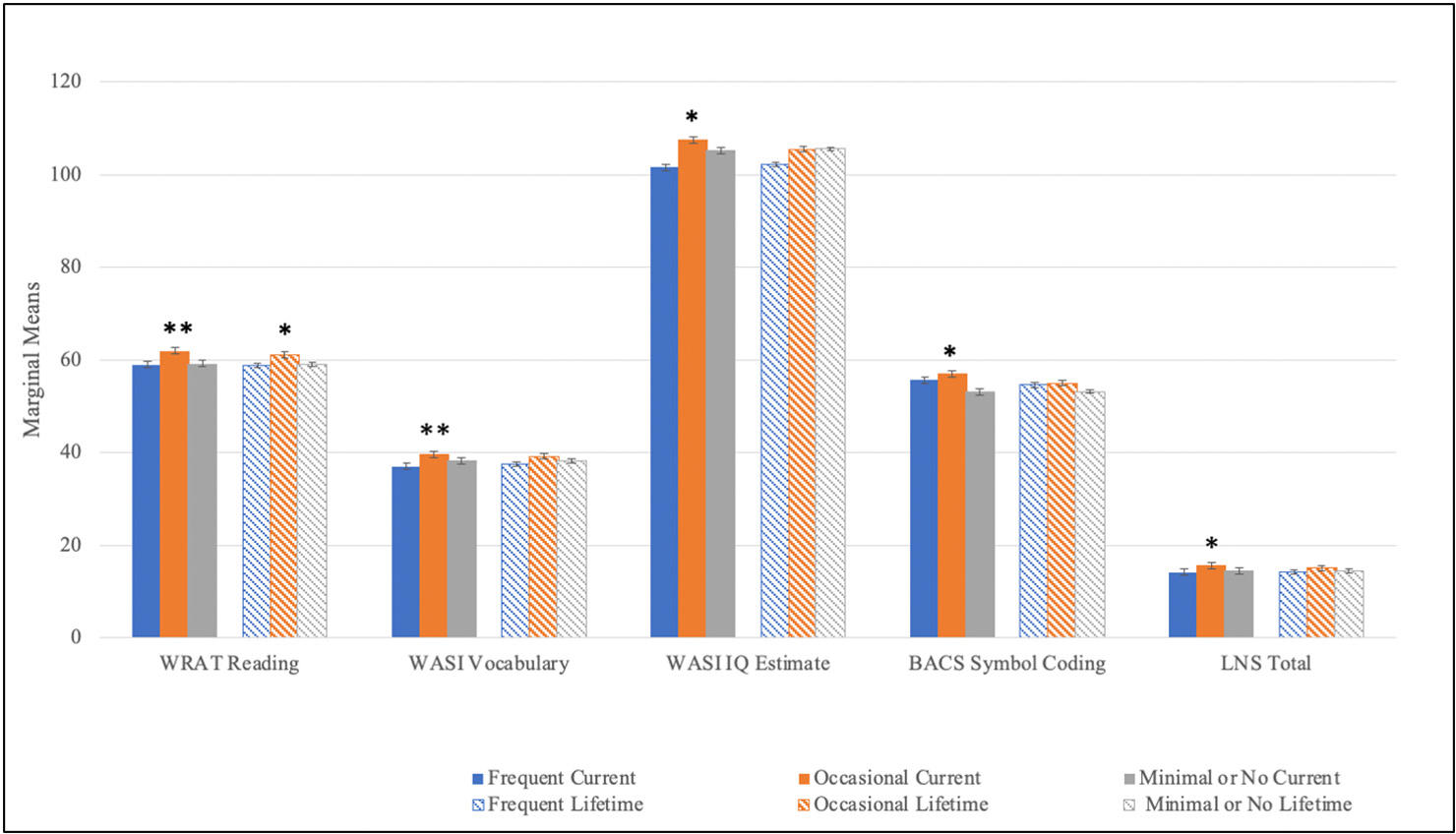
CHR Neurocognition by lifetime and current cannabis use-frequency. *p < .05, **p < .01. There was a significant main effect of lifetime cannabis use frequency on WRAT Reading Score (F (2,650) = 4.30, *p* = .01) using a Bonferroni corrected *p*-value = .02. Minimal Users scored lower than Occasional Users (Mean difference = −1.91, s.e. = 0.73, *p* = .01) and Occasional Users scored higher than Frequent users (Mean difference = 2.22, s.e. = .0.71 p = .01). There was also a significant main effect of current cannabis use frequency on WRAT Reading Score (F (2,651) = 5.90, *p* = .003) using a Bonferroni corrected *p*-value = .02. Minimal Users scored lower than Occasional users (Mean difference = −2.86, s.e. = 0.86, *p* < .001) and Occasional Users scored higher than Frequent users (Mean difference = 0.3.01, s.e. = 1.06 *p* = .004). There were trend level significant main effects of current cannabis use frequency on WASI Vocabulary (F (2,658) = 3.50, *p* = .03), WASI IQ Estimate (F (2,658) = 2.44, *p* = .09), LNS (F (2,652) = 3.69, *p* = .03), and BACS (F (2,666) = 2.88, *p* = .06) with a general pattern of Occasional users performing better than the Minimal to No Use groups (*p*s < .05) and in the case of the LNS, Occasional users scores significantly better than both the Frequent and the Minimal to No Use groups (*p*s = .01).WRAT Reading- Wide Range Achievement Test-Reading Subtest; WASI Vocabulary = Weschler Abbreviated Scale of Intelligence-Vocabulary Subtest; WASI IQ Estimate = Weschler Abbreviated Scale of Intelligence Full Scale Intelligence Quotient Estimate; BACS Symbol Coding = Brief Assessment of Cognition in Schizophrenia Symbol Coding Subtest; LNS Total = Letter Number Sequence Total Score.

**Fig. 5.**
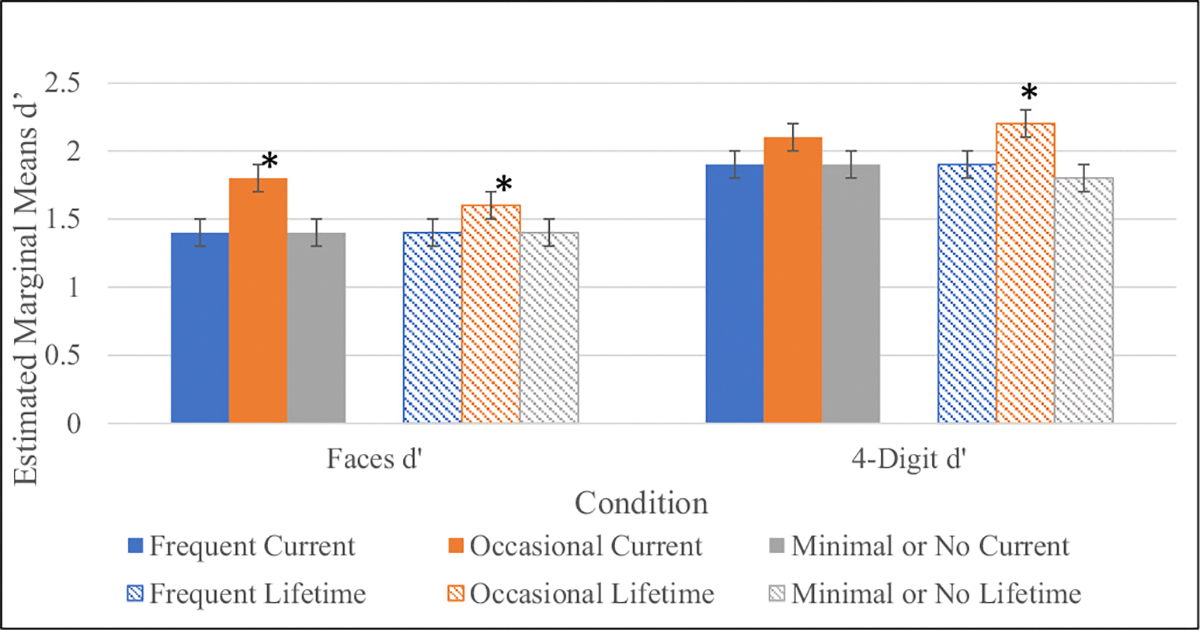
CHR Neurocognition by lifetime and current cannabis use-frequency-CPT-IP. *p < .05. There was a trend-level significant main effect of lifetime cannabis use frequency on CPT-IP 4 Digit Span (F (2,613) = 3.10, *p* = .05) with Occasional users displaying better performance than Minimal to No users (Mean difference = 0.34, s.e = 0.14, *p* = .01). There were also trend-level significant main effects of current cannabis use frequency on CPT-IP Faces (F (2,612) = 3.25, *p* = .04), with Occasional users displaying better performance than both Frequent (Mean difference = 0.34, s.e. = 0.17, *p* = .04) and Minimal to No users (Mean difference = 0.32, s.e. = 0.13, *p* = .01). CPT-IP = Continuous Performance Task-Identical Pairs.

**Fig. 6.**
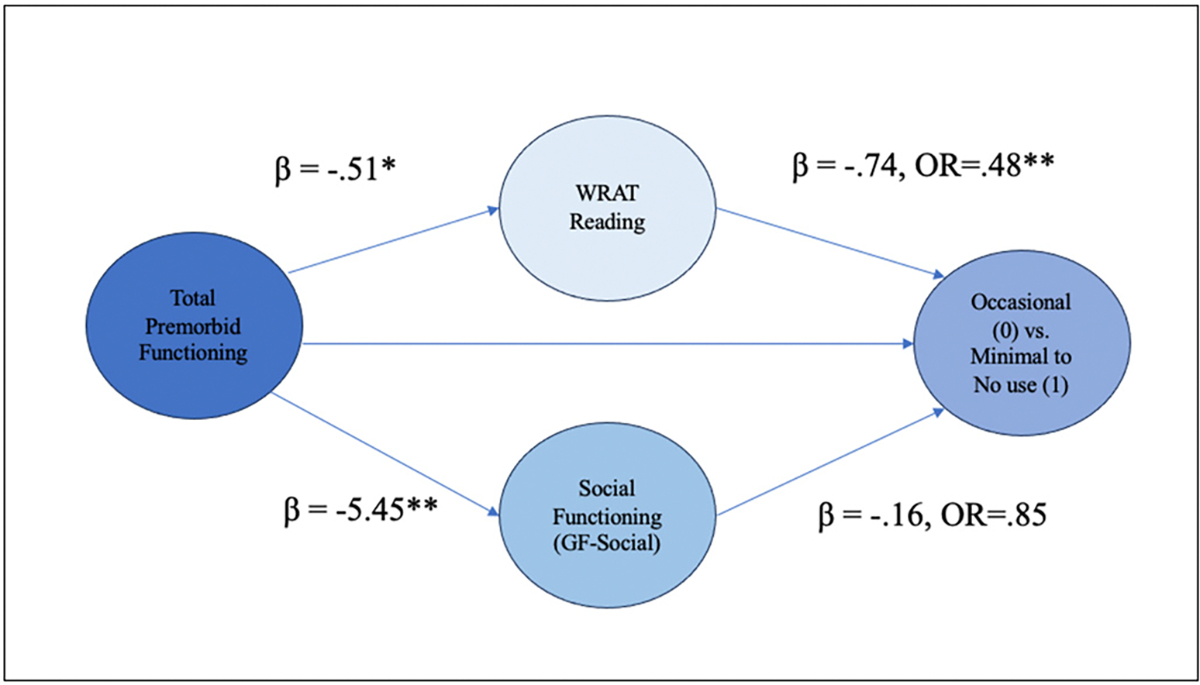
Path analysis of the relationship between total premorbid functioning and cannabis use frequency (Occasional vs. Minimal) with social functioning and WRAT Reading Score as mediators. ***p < .001, **p < .05, *p < .10. All significant and trend-level significant pathways are represented here. WRAT = Wide Range Achievement Test.

Individual univariate ANOVAs were employed to test the main effects of greatest current and lifetime cannabis use frequency pattern on neurocognitive performance. There was a statistically significant group and age main effect on WRAT-4 Reading Score using current cannabis use frequency as a grouping factor and a trend group main effect using lifetime cannabis use frequency as a grouping factor. No significant sex or interaction effects were observed for this test. Post-hoc tests revealed that occasional users displayed higher premorbid IQ compared to the other use-groups in both current and lifetime patterns (Fig. 4, Table 2). This pattern of better performance among occasional users was observed on tests of sustained attention and working memory at a trend level using current cannabis use pattern as a grouping factor (Figs. 5–6, Table 2). These trend level findings indicate that neurocognitive performance among CHR youth is associated with the frequency of use-reported at the time of testing, in this case specifically on tests of IQ, sustained attention, and working memory (Figs. 5–6, Table 2).

Follow up analyses added family income, age of onset of cannabis use, and cumulative use in the past 6 months as covariates along with alcohol use and tobacco use as additional within group factors. None of these variables significantly influenced the effect of cannabis use group on WRAT Reading score. Importantly however, significant main effects of some of these covariates and within person factors (family income, age of onset of cannabis use, alcohol use) nullified trend level effects previously observed of cannabis use group on WASI Vocabulary, WASI IQ Estimate, LNS, CPT-IP-Faces, and CPT-IP-4 Digit Span. Controlling for age, and sex, family income had a significant main effect on WASI Vocabulary (*F* = 17.68, *p* < .001), WASI IQ Estimate (*F* = 6.41, *p* = .01), LNS (*F* = 4.32, *p* = .04), CPT-IP- 4 Digit Span (*F* = 4.57, *p* = .03), and CPT-IP Faces (*F* = 3.25, *p* = .07, trend level). Age of onset of cannabis use had significant main effects on WASI Vocabulary (*F* = 8.44, *p* < .01), WASI IQ Estimate (*F* = 8.52, *p* < .01), and CPT-IP-4 Digit Span (*F* = 5.54, *p* = .02) and alcohol use had a significant (trend level) main effect on WASI Vocabulary (*F* = 3.39, *p* = .06). It was demonstrated in these analyses that each of these covariates/within person factors were associated with cannabis use frequency and had significant effects on neurocognition.

### 3.4. Structural equation models and path analyses exploring the relationship between premorbid functioning, social functioning, neurocognition and lifetime cannabis use frequency

**Fig. 7.**
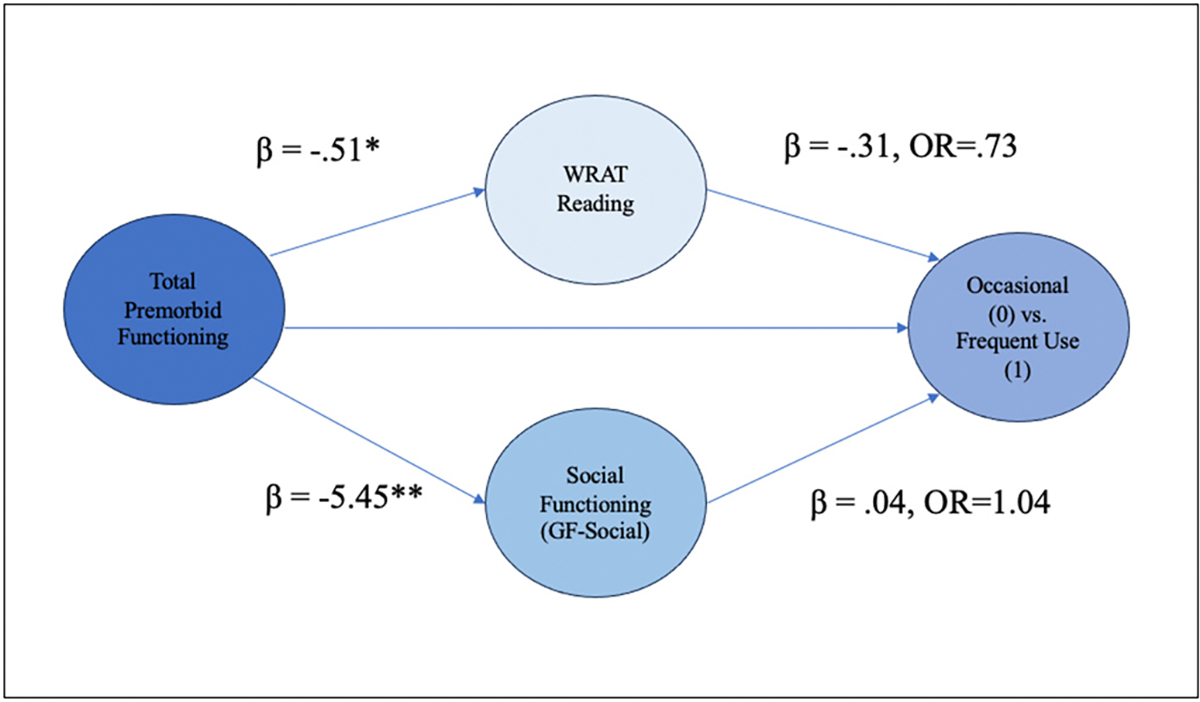
Path analysis of the relationship between total premorbid functioning and cannabis use frequency (Occasional vs. Frequent) with social functioning and WRAT Reading Score as mediators. ***p < .001, **p < .05, *p < .10. All significant and trend-level significant pathways are represented here. WRAT = Wide Range Achievement Test.

Assumptions of no/little multicollinearity were not violated, and full results of the binary logistic regression models including fit indices and sample sizes are outlined in Supplemental Tables 4–5, 6–7. The first path-analytic model explored the relationship between total premorbid functioning, social functioning, WRAT-4 Reading score and cannabis use frequency (occasional vs. minimal to no use) (Fig. 6; Supplementary Table 6). The target model was a good fit statistically. The direct effect from total premorbid functioning to cannabis use frequency was not statistically significant, however, the total effect was statistically significant, indicating that the combined indirect effects of social functioning and WRAT-4 Reading Score influence the relationship between total premorbid functioning and cannabis use frequency (occasional vs. minimal to no use). With respect to the first indirect effect, individuals who reported better total premorbid functioning (lower PAS scores) demonstrated higher premorbid IQ scores. Higher premorbid IQ in turn was associated with occasional use compared to minimal to no cannabis use frequency. With every 1 unit-increase in WRAT-4 Reading Score, the odds of going from an occasional user to a minimal to no user (reducing cannabis use frequency) decreased by 52 % (OR = 0.48, *p* < .001, 95 % CI [0.33–0.66]). Regarding the second indirect effect, total premorbid functioning was significantly associated with social functioning, and social functioning was associated with cannabis use frequency at trend level significance. This model overall suggests that premorbid IQ (high performance on the WRAT-4 Reading score) fully mediates the relationship between total premorbid functioning and cannabis use frequency (occasional vs. minimal to no use). A second model comparing the occasional vs. the frequent use group displayed sufficient model fit, but did not demonstrate a significant relationship between predictor variables and cannabis use frequency (Fig. 7, Supplementary Table 7).

## 4. Discussion

This study demonstrated evidence of better neurocognitive performance among CHR youth with occasional cannabis use patterns compared to those with frequent or minimal to no cannabis use (de la Serna et al., 2010; Rabin et al., 2011; Yücel et al., 2012). Specifically, occasional users performed significantly better on measures of premorbid IQ and showed a similar trend in sustained attention and working memory domains compared to frequent and minimal use groups. Occasional users also had a later age of onset of cannabis use compared to frequent users, and better premorbid, global, and social functioning compared to the other use-groups. The frequent use group demonstrated the most severe attenuated psychosis symptoms, followed by the occasional use group and the minimal to no use group. These findings overall suggest that better premorbid functioning may serve as a protective factor that predicts better neurocognition, social functioning and occasional cannabis use patterns, a relationship which has been theorized in the literature (de la Serna et al., 2010; Hanna et al., 2016; Jockers-Scherübl et al., 2007; Kumra et al., 2005; Ringen et al., 2013; Yücel et al., 2012; Zisook et al., 1992). Follow-up path analyses showed that cannabis use patterns are not directly predicted by premorbid functioning but are influenced indirectly by premorbid IQ. Better premorbid functioning is therefore associated with higher premorbid IQ that, in turn, is associated with a more occasional cannabis use pattern.

Critically, this study also incorporated family income (proxy for SES) as a covariate in follow-up analyses of neurocognition and found that across several neurocognitive tests, family income accounted for a more significant effect on neurocognition than cannabis use group. While there is substantial evidence across clinical populations of lower SES being associated with impairments in neurocognition (e.g. cognitive control, executive functioning), there are few studies that examine the impact of SES on cognition in psychosis (Amoretti et al., 2021; Buckley et al., 2019; Czepielewski et al., 2022; Duncan and Magnuson, 2012; Li et al., 2022). Age of onset of cannabis use also had a stronger effect on several neurocognitive tests compared to cannabis use group in this study, which is consistent with previous studies that have found that age of cannabis use initiation is a strong driver of neurocognition and brain development (Penzel et al., 2021; Pope et al., 2003; Scott et al., 2018). Additionally, alcohol and tobacco use have been reported to impact attenuated psychosis symptoms in this population and alcohol in particular was observed in this study to have a more significant effect on neurocognition than cannabis use group in a measure of vocabulary comprehension, indicating comorbid substance use is an important within person factor in the context of neurocognition (Auther et al., 2015; Buchy et al., 2014; Tapert et al., 2002; Ward et al., 2019).

The findings from the present study fit into an established yet heterogenous scientific framework surrounding cannabis use, neurocognition, and clinically significant psychosis. Several studies among individuals with established psychosis have demonstrated better cognitive performance and/or functioning among cannabis users compared to non-users (Ferraro et al., 2019; Menendez-Miranda et al., 2019; Potvin et al., 2008; Rabin et al., 2011; Schnakenberg Martin et al., 2016; Schnell et al., 2009; Schoeler et al., 2016; Yücel et al., 2012; Zisook et al., 1992). Conversely, some studies have demonstrated no observable association between cannabis use and cognition in schizophrenia (Bahorik et al., 2014; Mata et al., 2008). Ferraro et al. (2020) delineated cannabis use groups by never, occasional, and daily users among an FEP cohort and found that both occasional FEP users and controls had higher IQ compared to the never or daily use groups in both clinical groups. Importantly, these investigators found that daily users in both clinical groups had lower academic premorbid functioning than occasional users and individuals with no use, and had higher social functioning than non-users (comparison with occasional users was non-significant) (Ferraro et al., 2020). While the exact interpretation of these disparate cannabis/neurocognition findings is the subject of some debate in the psychosis literature, researchers have posited that individuals who can maintain substance use behaviors, which require social and global functioning skills, may also have better neurocognitive abilities and premorbid functioning in comparison to non-users (Dellazizzo et al., 2023; Rabin et al., 2011; Rodríguez-Sancheź et al., 2010; Schnell et al., 2009).

The association between higher premorbid functioning, better neurocognition, and better social functioning and its relationship to cannabis use behaviors has been investigated in case-control examples in psychosis cohorts but has been less explored in CHR (Ferraro et al., 2019; Zisook et al., 1992). The occasional use pattern observed in this study may be reflective of the protective nature of better premorbid functioning and associated neurocognition and social functioning. Given that subjects in this study with frequent use performed worse in these domains than the occasional use group, it is possible that CHR cannabis users with higher functional and neurocognitive profiles may be less likely to use cannabis in excess. Elucidating the role of premorbid functioning in the later formation of one’s cannabis use pattern may help provide an alternative conceptualization as opposed to any causal notion that a certain level of cannabis use “improves” neurocognitive performance. Premorbid functioning and neurocognition appear to be the primary drivers of cannabis use frequency rather than the inverse. This is an important point, given that there is no evidence to our knowledge to support the likelihood of improvement in cognition and/or functioning among CHR individuals with minimal/no use who begin using cannabis at a more regular frequency. Although potential therapeutic benefits of tetrahydrocannabinol (THC), the primary psychoactive compound in cannabis, have been studied across a range of medical conditions (eg, spasticity in Multiple Sclerosis, chronic pain, anorexia in cancer) (Jones and Vlachou, 2020; Legare et al., 2022; Petzke et al., 2022) there is no evidence beyond select preclinical models (Calabrese and Rubio-Casillas, 2018; Sarne, 2019) of enhancement in human cognition or functioning in any clinical population. A recent systematic review by Wieghorst et al. (2022) found that across various clinical contexts, low levels of exposure to THC was associated with occasional albeit short-term cognitive impairments, however the risk for worsening of cognitive deficits appeared to increase with higher exposure to THC in some studies (Wieghorst et al., 2022). However, several studies have begun to explore the potential role of cannabidiol (CBD), a nonpsychoactive compound derived from cannabis, on neurocognition and clinical symptoms in early psychosis with the hypothesis that CBD may provide antipsychotic, anti-inflammatory and neuroprotective benefits (Bonaccorso et al., 2019; Chesney et al., 2022; Davies and Bhattacharyya, 2019; Dixon and Cadenhead, 2023; Leweke et al., 2021).

### 4.1. Limitations

Due to the cross-sectional design of this study, longitudinal analyses of cannabis use frequency, neurocognition, and functioning were not conducted, limiting our understanding of how observed trends may change over time. This is especially important in the context of the later age of onset of cannabis use observed among occasional users. Information on lifetime cannabis use patterns, social functioning, and premorbid functioning were also limited by self-report. Possible strategies to glean more information about multi-dimensional premorbid functioning domains would be to employ principal component analyses or factor analyses as was demonstrated by Ferraro et al. (2020). Lifetime cannabis use patterns may also have been a high estimate of actual use-frequency, as this variable was derived from highest reported lifetime use pattern and did not control for a possible reduction in use-frequency. Additionally, cumulative use (past 6 months) was not truly reflective of lifetime cumulative use. Regarding family income as a proxy covariate for SES, this is a one-dimensional measure that does not capture other critical determinants of SES. Future studies may choose to employ measurements of SES that reflect several domains such as the CAPSES which measures material capital, human capital, and social capital (Oakes and Rossi, 2003). While the structural equation models/path analyses demonstrated good model fit and highlighted important relationships between premorbid functioning, neurocognition, and cannabis use frequency, it is important they be interpreted with caution. As these were basic follow-up exploratory models, there are undoubtedly other variables and latent constructs not included in these models that may also contribute to the variance in later cannabis use frequency among CHR youth.

### 4.2. Conclusions

This study contributed to emerging studies in CHR and provided parallel findings to existing evidence from both FEP and chronic psychosis populations of better neurocognitive performance among those who use cannabis, with the important distinction that occasional use (monthly to yearly) versus frequent use (daily to weekly) appears to drive this phenomenon. Importantly however, any effects of neurocognition among occasional users we believe is not reflective of any protective feature of cannabis itself but rather these individuals represent higher functioning CHR youth with better premorbid functioning.

While the global discourse regarding the role of cannabis use among youth at risk for psychosis may be mixed in its findings, continued attention to the public health significance of high-rates of use in young people at CHR is warranted (Colizzi and Murray, 2018; Penzel et al., 2022). Repeated cannabis use increases the likelihood of an acute psychiatric event and researchers warn of the lack of consistent health data on the high-potency THC cannabis that is widely circulated among today’s youth (Murray and Hall, 2020). Future studies should aim to collect multi-dimensional data on cannabis use in young people at risk for severe mental illness to better understand the nuances of why this population uses cannabis at such elevated rates, and how cannabis use broadly affects health, functioning, and psychiatric symptoms over time.

## Supplementary Material

supplementary material

## Figures and Tables

**Table 1:** Baseline CHR demographics by cannabis use frequency.

CHR (*N* = 698)	3. Frequent use N (%) 165 (23.2)	2. Occasional use N (%) 127 (17.9)	1. Minimal or no use N (%) 406 (57.2)	Test-statistic	p-Value	Post hoc tests

	M (SD)	M (SD)	M (SD)	F-statistic	p	Groups
Age (years)	20 (3.89)	19.59 (3.56)	17.02 (3.87)	46.03	<.001**	1vs 2** 1vs 3** 2 vs 3
Years of education	11.98 (3.31)	12.20 (3.77)	10.17 (3.49)	25.12	<.001**	1vs 2** 1vs 3** 2 vs 3
Age of first cannabis use	15.51 (2.60)	16.33 (2.40)	16.81 (3.01)	6.92	.001*	1vs 2 1vs 3** 2 vs 3*
Family income level	4.04 (1.81)	4.23 (1.79)	4.51 (1.74)	3.47	.03*	1 vs 2 1 vs 3* 2 vs 3
Cumulative use of cannabis in past 6 months	74.38 (65.52)	13.19 (29.43)	0.71 (0.81)	82.79	<.001**	1 vs 2 1 vs 3** 2 vs 3**
	N (%)	N (%)	N (%)	*χ* ^2^	p	
Sex				14.34	<.001**	
% male	111 (29.21)	62 (16.31)	205 (53.94)			
Alcohol use				145.82	<.001**	
% use	103 (40.90)	78 (31.0)	71 (28.2)	149.13	<.001**	
No use	62 (37.60)	49 (38.60)	335 (82.50)			
1–4 × per month	63 (38.20)	50 (39.40)	53 (13.10)			
1–4 × per week	37 (22.40)	26 (20.50)	16 (3.90)			
Daily	3 (1.80)	2 (1.60)	2 (0.50)			
Tobacco use				148.36	<.001**	
% use	75 (57.70)	39 (30.0)	16 (12.30)	156.75	<.001**	
No use	90 (54.00)	88 (69.30)	390 (96.10)			
Occasional	41 (24.80)	29 (22.80)	6 (1.50)			
>1 time per day	34 (20.6)	10 (7.90)	10 (2.50)			

** p <.001.

* p <.05.

**Table 2:** CHR baseline neurocognition by lifetime and current cannabis use frequency.

	3. Frequent use	2. Occasional use	1. Minimal to no use	F-statistic	Bonferroni p (.004)	Group comp.
	M (SD)	M (SD)	M (SD)			

WRAT Reading-Lifetime	58.83 (0.60)	61.05 (0.62)	59.14 (0.35)	4.30	.01*	1 vs 2**
Age				92.66	<.001***	1 vs 3
Sex				0.19	.67	2 vs 3**
df (2, 650)						
WRAT Reading-Current	59.98 (7.19)	63.19 (5.58)	58.81 (7.46)	5.90	.003**	1 vs 2**
Age				99.60	<.001***	1 vs 3
Sex				0.20	.66	2 vs 3**
df (2, 651)						
WASI Vocab-Lifetime	38.52 (6.36)	40.03 (6.37)	37.57 (6.69)	0.49	.61	
Age				76.40	<.001***	
Sex				0.41	.52	
df (2, 650)						
WASI Vocab-Current	37.83 (6.61)	40.64 (6.70)	37.97 (6.55)	3.50	.03	1 vs 2*
Age				82.94	<.001***	1 vs 3*
Sex				0.02	.89	2 vs 3**
df (2, 650)						
WASI Matrix Reas.- Lifetime	20.85 (4.20)	20.85 (5.19)	20.73 (4.56)	0.46	.61	
Age				11.48	<.001***	
Sex				0.83	.36	
df (2, 650)						
WASI Matrix Reas.-Current	20.44 (4.15)	21.15 (4.96)	20.79 (4.63)	0.85	.43	
Age				11.79	<.001***	
Sex				0.55	.46	
df (2, 655)						
WASI FSIQ Est.- Lifetime	103.14 (16.39)	106.02 (17.16)	105.17 (16.75)	2.10	.13	
Age				4.91	.03*	
Sex				1.64	.20	
df (2, 657)						
WASI FSIQ Est.-Current	102.25 (15.87)	107.63 (17.64)	104.99 (16.74)	2.44	.09*	1 vs 2
Age				4.01	.05	1 vs 3*
Sex				0.49	.48	2 vs 3*
df (2, 657)						
HVLT-Lifetime	25.82 (4.86)	26.48 (5.20)	26.45 (5.31)	1.11	.33	
Age				10.86	.001**	
Sex				11.18	<.001***	
df (2, 648)						
HVLT-Current	25.60 (5.31)	27.14 (4.35)	26.34 (5.30)	1.33	.27	
Age				9.02	.003**	
Sex				6.69	.01*	
df (2, 648)						
BACS-Lifetime	55.46 (15.03)	55.79 (14.57)	52.54 (14.65)	0.80	.21	
Age				14.83	<.001***	
Sex				3.62	.19	
df (2, 665)						
BACS-Current	56.12 (13.17)	57.91 (13.67)	52.73 (15.09)	2.88	.06*	1 vs 2*
Age				15.92	<.001***	1 vs 3
Sex				1.37	.24	2 vs 3
df (2, 666)						
LNS-Lifetime	14.74 (4.10)	15.43 (3.50)	14.18 (3.80)	1.57	.45	
Age				40.92	<.001***	
Sex				1.70	.06	
df (2, 651)						
LNS-Current	14.47 (4.01)	16.07 (3.39)	14.34 (3.55)	3.69	.03*	1 vs 2**
Age				44.02	<.001***	1 vs 3
Sex				0.38	.54	2 vs 3**
df (2, 652)						
CPT-IP Faces d-prime-Lifetime	1.48 (0.91)	1.67 (0.93)	1.34 (1.10)	2.08	.13	
Age				15.84	<.001***	
Sex				2.09	.15	
df (2, 611)						
CPT-IP Faces d-prime-Current	1.48 (0.88)	1.80 (1.03)	1.37 (1.05)	3.25	.04*	
Age				16.90	<.001***	
Sex				0.31	.58	
df (2, 612)						
CPT-IP 4-Digit d-prime-Lifetime	2.12 (1.33)	2.23 (1.49)	1.70 (1.24)	3.10	.05*	
Age				59.20	<.001***	
Sex				0.01	.91	
df (2.613)						
CPT-IP 4-Digit d-prime-Current	2.12 (1.27)	2.29 (1.46)	1.82 (1.31)	1.08	.34	
Age				69.79	<.001***	
Sex				0.57	.45	
df (2.614)						
CPT-IP Shapes d-prime-Lifetime	2.29 (1.50)	2.14 (1.34)	2.00 (1.42)	0.54	.58	
Age				4.64	.03*	
Sex				0.01	.91	
df (2, 613)						
CPT-IP Shapes d-prime-Current	2.17 (1.45)	2.37 (1.45)	2.04 (1.42)	1.00	.37	
Age				5.41	.02*	
Sex				0.02	.88	
df (2, 612)						
Seidman CPT Total QA% Hits-Lifetime	93.62 (15.63)	92.86 (18.82)	92.69 (14.52)	0.39	.67	
Age				14.16	<.001***	
Sex				8.31	.004**	
df (2, 643)						
Seidman CPT Total QA% Hits-Current	94.69 (12.57)	91.99 (22.13)	92.76 (14.98)	1.08	.34	
Age				14.30	<.001***	
Sex				12.51	<.001***	
Sex * Group				3.07	.05	
df (2, 644)						
Seidman CPT Total QA3% Hits-Lifetime	79.91 (17.42)	79.64 (22.39)	78.66 (17.60)	0.09	.92	
Age				13.96	<.001***	
Sex				6.79	.01*	
df (2, 644)						
Seidman CPT Total QA3% Hits-Current	79.83 (16.53)	79.22 (22.55)	70.03 (18.22)	0.21	.81	
Age				14.93	<.001***	
Sex				13.43	<.001***	
Sex * Group				5.66	.004**	
df (2, 645)						
Seidman CPT Total QA1AINT% Hits-Lifetime	76.94 (18.91)	74.24 (21.71)	73.95 (18.69)	0.09	.91	
Age				16.47	<.001***	
Sex				0.02	.88	
df (2, 649)						
Seidman CPT Total QA1AINT% Hits-Current	76.77 (18.01)	75.91 (23.07)	73.38 (18.97)	0.05	.96	
Age				17.16	<.001***	
Sex				0.71	.40	
Sex * Group				3.10	.05	
df (2, 644)						

*** *p* < .001.

** *p* < .004.

* *p* < .09 (trend-level significance).
