# Supplementary material for: Occasional cannabis use is associated with higher premorbid functioning and IQ in youth at clinical high-risk (CHR) for psychosis: Parallel findings to psychosis cohorts
Source: Schizophr Res. Author manuscript; Available in PMC 2025 Mar 31. (PMC11955428; doi:10.1016/j.schres.2024.07.032)
Supplement: supplementary material [file NIHMS2065178-supplement-supplementary_material.docx]

**Supplementary Section 1.**

Given that cannabis-use frequency is a 3-level ordinal variable, ordinal linear regression and multinomial linear regressions were considered. These methods were limited however in their ability to capture information specifically about occasional use patterns. Ordinal linear regressions would only be sufficient in discussing how a one-unit increase in neurocognitive score or social functioning increased or decreased the likelihood of being a heavier or lower frequency cannabis-user (comparing minimal to occasional and Frequent, Frequent and occasional to minimal) (*Ordinal Logistic Regression*). Multinomial linear regression would treat each cannabis-use group as unrelated nominal variables, which would not capture the relatedness of the cannabis-use groups as they are ordinal in nature (*Multinomial Logistic Regression*).

**Supplementary Table 1. CHR SOPS domains by cannabis-use frequency**

|  | Frequent Use | Occasional Use | Minimal to No Use | Test- Statistic | p-value | Effect size | | Post Hoc Tests | | |  |
| --- | --- | --- | --- | --- | --- | --- | --- | --- | --- | --- | --- |
|  | M(SD) | M(SD) | M (SD) | F-statistic | *p* | *η^2^* | Group Comp. | Mean Diff. | Std.Err | | *p* |
|  |  |  |  |  |  |  |  |  |  |  | |
| *SOPS*  *Positive*  *_df (2,694)_* | 13.56(3.55) | 12.40 (3.48) | 12.81 (3.30) | 4.62 | .01* | .01 | 1 vs 2  1 vs 3  2 vs 3 | .39  -.77  -1.16 | .35  .33  .40 | .28  .02*  .004* | |
| *P1- Unusual Thought Content* | 3.85 (1.0) | 3.7 (1.05) | 3.7 (1.14) | 1.01 | .33 |  |  |  |  |  | |
| *P2-Suspiciousness/Persecutory Ideas* | 3.11 (1.38) | 2.87 (1.48) | 3.15 (1.43) | 1.95 | .14 |  |  |  |  |  | |
| *P3- Grandiosity* | 1.35 (1.47) | .98 (1.35) | .72 (1.21) | 13.64 | <.001** | .04 | 1 vs 2  1 vs 3  2 vs 3 | -.25  -.62  -.37 | .13  .12  .15 | .06  <.001**  .02* | |
| *P4- Perceptual Abnormalities* | 3.11 (1.37) | 2.81 (1.55) | 3.23 (1.36) | 4.34 | .01* | .01 | 1 vs 2  1 vs 3  2 vs 3 | .42  .12  -.30 | .14  .13  .17 | .003*  .35  .07 | |
| *P5- Disorganized Communication* | 2.15 (1.53) | 2.03 (1.44) | 2.01 (1.50) | .50 | .61 |  |  |  |  |  | |
| *SOPS Negative*  *_df (2,685)_*  *SOPS Disorganized*  *_df(2,685)_* | 11.90 (5.76)  5.19 (2.91) | 11.58 (6.33)  4.94 (3.20) | 12.45 (6.56)  5.33 (3.34) | 1.85  .97 | .16  .38 | .01  .00 |  | . |  |  | |
| *SOPS General*  *_df (2, 684)_* | 9.78 (4.10) | 8.52 (3.91) | 9.60 (4.42) | 4.11 | .02* | .01 | 1 vs 2  1 vs 3  2 vs 3 | 1.21  -.03  -1.24 | .45  .42  .51 | .01*  .95  .02* | |

**^*p<.05, **p<.001^**

**Table 2. CHR global, social, and role functioning by cannabis-use frequency**

|  | Frequent Use | Occasional Use | Minimal or No Use | Test-Statistic | p-value | Effect size | Post Hoc Tests | | | |
| --- | --- | --- | --- | --- | --- | --- | --- | --- | --- | --- |
|  | M(SD) | M(SD) | M (SD) | F-statistic | p-value | *η^2^* | Group Com. | Mean Diff. | Std.Err | *p* |
| *GAF df (2,693)* | 51.07(11.10) | 53.36(11.51) | 49.98(12.44) | 3.87 | .02* | 0.01 | 1 vs 2 | -3.38 | 1.22 | .01* |
|  |  |  |  |  |  |  | 1vs 3 | -1.1 | 1.11 | 0.33 |
|  |  |  |  |  |  |  | 2 vs 3 | 2.29 | 1.42 | 0.11 |
| *GF-Role df (2,694)* | 6.08(2.10) | 6.30 (2.34) | 6.22 (2.21) | 0.33 | 0.72 | 0 |  |  |  |  |
|  |  |  |  |  |  |  |  |  |  |  |
| *GF-Social df (2,694)* | 6.64 (1.38) | 6.75 (1.55) | 6.21 (1.54) | 12.29 | <.001** | 0.03 | 1 vs 2 | -0.65 | 0.16 | <.001** |
|  |  |  |  |  |  |  | 1vs 3 | -0.56 | 0.15 | <.001** |
|  |  |  |  |  |  |  | 2 vs 3 | 0.09 | 0.18 | 0.6 |

^**^*^p^*^<.001^

**Supplementary Table 3. CHR Premorbid functioning domains by lifetime cannabis-use frequency**

|  | Frequent Use | Occasional  Use | | | Minimal to No Use | Test- Statistic | p-value | Effect-size | Post Hoc Tests | | | | | |
| --- | --- | --- | --- | --- | --- | --- | --- | --- | --- | --- | --- | --- | --- | --- |
|  | M (SD) | M(SD) | M(SD) | | | F-statistic | *p* | *η^2^* | Group Comp. | Mean Diff. | | Std.Err | | *p* |
| *PAS Childhood*  *_df (2,684)_* | .24(.16) | .23 (.17) | | .25 (.17) | | .77 | .46 |  |  |  |  | |  | |
| *PAS Early Adolescent*  *_df (2,679)_* | .29 (.15) | .27 (.14) | | .32 (.18) | | 2.19 | .11 |  |  |  |  | |  | |
| *PAS Late Adolescent*  *_df (2,487)_* | .30 (.16) | .25 (.15) | | .32 (.21) | | 5.61 | .004* | .02 | 1 vs 2  1 vs 3  2 vs 3 | .07  .01  -.06 | .02  .02  .02 | | .001*  .02*  .01* | |
| *PAS Adult*  *_df (2,253)_* | .29 (.17) | .25 (.20) | | .39 (.25) | | 11.35 | <.001** | .08 | 1 vs 2  1 vs 3  2 vs 3 | .15  .11  -.04 | .03  .03  .04 | | <.001**  <.001**  .29 | |
| *PAS Total*  *_df (2,685)_* | .28 (.12) | .25(.12) | | .30 (.16) | | 4.15 | .02* | .01 | 1 vs 2  1 vs 3  2 vs 3 | .04  .02  -.03 | .02  .01  .02 | | .004*  .30  .09 | |

^*^*^p^*^<.05^

^**^*^p^*^<.001^

**Table 4. Collinearity diagnostics for dependent variable comparing Occasional cannabis use to Frequent cannabis use**

**Table 5. Collinearity diagnostics for dependent variable comparing Occasional cannabis use to Minimal to No cannabis use**

**Supplemental Tables 6-7. Binary Logistic Regression Results**

**Table 6.**

| **Binary Logistic Regression- Occasional compared to Minimal to No Use-WRAT Reading** |  |
| --- | --- |
| **MODEL FIT INFORMATION** | Occasional N=125; Minimal to No use N=403  *N*=528 |
| Number of Free Parameters | 10 |
| Chi Square Test of Model Fit | 4.31 |
| Degrees of Freedom | 1 |
| P-Value | .04 |
| RMSEA | 0.07 |
| CFI | .98 |
| TLI | .93 |

| **MODEL RESULTS** |  |  |  |  |  |  |
| --- | --- | --- | --- | --- | --- | --- |
| Occasional vs. Minimal to No use- WRAT Reading |  |  |  |  |  |  |
| ON | ESTIMATE | S.E. | Est./S.E. | P-Value | 95% CI Lower | 95% CI Upper |
| MIN |  |  |  |  |  |  |
| WRAT | -.74 | .18 | -.41 | <.001 |  |  |
| SF | -.16 | .10 | -1.71 | .09 |  |  |
| WRAT |  |  |  |  |  |  |
| Total Premorbid Functioning | -.51 | .23 | -2.23 | .03 |  |  |
| SF |  |  |  |  |  |  |
| Total Premorbid Functioning | -5.45 | .33 | -16.49 | <.001 |  |  |
| Intercepts |  |  |  |  |  |  |
| SF | 7.99 | .11 | 75.55 | <.001 |  |  |
| WRAT | .20 | .07 | 2.84 | .01 |  |  |
| Thresholds |  |  |  |  |  |  |
| MIN1 | -2.05 | .81 | -2.5 | .01 |  |  |
| Residual Variances |  |  |  |  |  |  |
| SF | 1.66 | .09 | 19.25 | <.001 |  |  |
| WRAT | .67 | .04 | 15.47 | <.001 |  |  |
| Logistic Regression Odds Ratio Results |  |  |  |  |  |  |
| MIN |  |  |  |  |  |  |
| WRAT | .48 | .09 |  |  | .33 | .66 |
| SF | .85 | .08 |  |  | .70 | 1.02 |
| R-Square Observed Variable | ESTIMATE | S.E. | Est./S.E. | P-Value |  |  |
| SF | .27 | .03 | 9.35 | <.001 |  |  |
| WRAT | .01 | `.01 | 1.05 | .29 |  |  |
| Total, Total Indirect, Specific Indirect and Direct Effects | ESTIMATE | S.E. | Est./S.E. | P-Value |  |  |
| Effects from Total Premorbid Functioning to MIN |  |  |  |  |  |  |
| Total | 2.35 | .74 | 3.19 | .001 |  |  |
| Total Indirect | 1.27 | .55 | 2.32 | .02 |  |  |
| Specific Indirect Effect 1 (Premorbid Functioning- Social functioning- MIN) | .07 | .04 | 1.74 | .08 |  |  |
| Specific Indirect Effect 2 (Premorbid Functioning- WRAT- MIN) | .03 | .01 | 1.97 | .05 |  |  |
| Direct (Premorbid Functioning to MIN) | .08 | .06 | 1.24 | .22 |  |  |

**Table 7.**

| **Binary Logistic Regression- Occasional compared to Frequent Use-WRAT Reading** |  |
| --- | --- |
| **MODEL FIT INFORMATION** | Occasional N=125; Frequent use N=161  *N*=286 |
| Number of Free Parameters | 10 |
| Chi Square Test of Model Fit | 4.31 |
| Degrees of Freedom | 1 |
| P-Value | .04 |
| RMSEA | .07 |
| CFI | .99 |
| TLI | .92 |

| **MODEL RESULTS** |  |  |  |  |  |  |
| --- | --- | --- | --- | --- | --- | --- |
| Occasional vs. Frequent use- WRAT Reading |  |  |  |  |  |  |
| ON | ESTIMATE | S.E. | Est./S.E. | P-Value | 95% CI Lower | 95% CI Upper |
| Frequent |  |  |  |  |  |  |
| WRAT | -.31 | .18 | -1.78 | .08 |  |  |
| SF | .04 | .10 | .38 | .71 |  |  |
| WRAT |  |  |  |  |  |  |
| Total Premorbid Functioning | -.51 | .23 | -2.23 | .03 |  |  |
| SF |  |  |  |  |  |  |
| Total Premorbid Functioning | -5.45 | .33 | -16.49 | <.001 |  |  |
| Intercepts |  |  |  |  |  |  |
| SF | 7.99 | .11 | 75.55 | <.001 |  |  |
| WRAT | .20 | .01 | 2.84 | <.001 |  |  |
| Thresholds |  |  |  |  |  |  |
| Frequent1 | .41 | .83 | .50 | .62 |  |  |
| Residual Variances |  |  |  |  |  |  |
| SF | 1.66 | .09 | 19.35 | <.001 |  |  |
| WRAT | .67 | .04 | 15.47 | <.001 |  |  |
| Logistic Regression Odds Ratio Results |  |  |  |  |  |  |
| Frequent |  |  |  |  |  |  |
| WRAT | .73 | .13 |  |  | .51 | 1.00 |
| SF | 1.04 | .10 |  |  | .86 | 1.25 |
| R-Square Observed Variable | ESTIMATE | S.E. | Est./S.E. | P-Value |  |  |
| SF | .27 | .03 | 9.37 | <.001 |  |  |
| WRAT | .01 | `.01 | 1.05 | .29 |  |  |
| Total, Total Indirect, Specific Indirect and Direct Effects | ESTIMATE | S.E. | Est./S.E. | P-Value |  |  |
| Effects from Total Premorbid Functioning to Frequent |  |  |  |  |  |  |
| Total | 1.83 | 1.09 | 1.67 | .10 |  |  |
| Total Indirect | -.04 | .53 | -.07 | .94 |  |  |
| Specific Indirect Effect 1 (Premorbid Functioning- Social functioning- Frequent) | -.20 | .53 | -.37 | .71 |  |  |
| Specific Indirect Effect 2 (Premorbid Functioning- WRAT- Frequent) | .16 | .12 | 1.29 | .20 |  |  |
| Direct (Premorbid Functioning to Frequent) | 1.86 | 1.19 | 1.56 | .12 |  |  |

The above path-analytic model was tested to explore the relations between total premorbid functioning, social functioning, WRAT Reading score, and cannabis-use frequency (Occasional vs. Frequent use) (**Figure 6; Supplementary Table 7**). The target model was again a good fit statistically based on RMSEA and CFI fit indices however was not a good fit based on Chi-Square test of model fit. In this model, neither the direct effect between total premorbid functioning and cannabis-use frequency nor the total effect of the model was statistically significant. Of the two indirect effects, neither compound path was significant. Relations observed in the third model between total premorbid functioning, social functioning, and WRAT Reading Score remained the same, however neither WRAT Reading score nor social functioning predicted cannabis-use frequency (Occasional vs. Frequent use) in this model.

References

*Multinomial Logistic Regression | R Data Analysis Examples*. (n.d.). Retrieved June 30, 2023, from https://stats.oarc.ucla.edu/r/dae/multinomial-logistic-regression/

*Ordinal Logistic Regression | R Data Analysis Examples*. (n.d.). Retrieved June 30, 2023, from https://stats.oarc.ucla.edu/r/dae/ordinal-logistic-regression/
